# Evidence for regulatory diversity and auto-regulation at the TAC1 locus in sensory neurones

**DOI:** 10.1186/1742-2094-8-10

**Published:** 2011-02-04

**Authors:** Lynne Shanley, Marissa Lear, Scott Davidson, Ruth Ross, Alasdair MacKenzie

**Affiliations:** 1 School of Medical Sciences, University of Aberdeen, AB25 2ZD, UK

## Abstract

The neuropeptide substance-P (SP) is expressed from the TAC1 gene in sensory neurones where it acts as a key modulator of neurogenic inflammation. The promoter of TAC1 (TAC1prom) plays a central role in the regulation of the TAC1 gene but requires the presence of a second regulatory element; ECR2, to support TAC1 expression in sensory neurones and to respond appropriately to signalling pathways such as MAPkinases and noxious induction by capsaicin. We examined whether the effect of capsaicin on ECR2-TAC1prom activity in larger diameter neurones was cell autonomous or non- cell autonomous. We demonstrate that TRPV1 is not expressed in all the same cells as SP following capsaicin induction suggesting the presence of a non-cell autonomous mechanism for TAC1 up-regulation following capsaicin induction. In addition, we demonstrate that induction of SP and ECR1-TAC1prom activity in these larger diameter neurones can be induced by potassium depolarisation suggesting that, in addition to capsaicin induction, transgene activity may be modulated by voltage gated calcium channels. Furthermore, we show that NK1 is expressed in all SP- expressing cells after capsaicin induction and that an agonist of NK1 can activate both SP and the transgene in larger diameter neurones. These observations suggest the presence of an autocrine loop that controls the expression of the TAC1 promoter in sensory neurones. In contrast, induction of the TAC1 promoter by LPS was not dependent on ECR2 and did not occur in large diameter neurones. These studies demonstrate the diversity of mechanisms modulating the activity of the TAC1 promoter and provide novel directions for the development of new anti-inflammatory therapies.

## Introduction

The neuropeptide substance P (SP), encoded by the Tachykinin 1 (TAC1) gene, acts as a key regulator of neurogenic inflammation [1,2]. Consistent with this role SP is usually expressed in nociceptive C-fibre sensory neurones within the dorsal root ganglia (DRG) [2]. In addition, it has been established that the expression of SP is up-regulated in larger diameter A and B fibre sensory neurones following noxious stimulation, an expression pattern also associated with the development of inflammatory pain and hyperalgesia [3,4]. Therefore, gaining a better understanding of the mechanisms involved in controlling the expression of the TAC1 gene in sensory neurones will be critical to understanding the mechanisms responsible for exacerbating the distressing symptoms of many forms of chronic inflammatory disease.

TAC1 gene expression, in common with the inflammatory pain response, is modulated by a number of different stimuli such as bacterial infection and noxious stimulation that often results in depolarisation of sensory neurones. Accepted paradigms for each of these processes include bacterial wall lipopolysaccharide (LPS) [5,6] capsaicin [7-10] and potassium-induced cell depolarisation that have all been shown to modulate TAC1 promoter (TAC1prom) activity [11,12]. Because of the critical role that TAC1 plays in neurogenic inflammation we explored whether there was any commonality in the mechanisms that influence the activity of TAC1prom in different populations of sensory neurones following stimulation by capsaicin, LPS or potassium.

In addition to noxious stimulation capsaicin treatment has been widely used as a model system in the study of hyperalgesia [13,14]. Capsaicin activates the transient receptor potential cation channel, subfamily V, member 1 (TRPV1) a major integrator of noxious stimuli that is expressed on a subset of SP expressing DRG neurones [7-10]. Capsaicin has also been shown to induce expression of TAC1 in vivo [9,10] and gene deletions of the SP-receptor (NK1) significantly reduces the hyperalgesic affects of capsaicin [15]. We have previously shown that capsaicin could induce the activity of TAC1prom but only in the presence of the evolutionary conserved region 2 (ECR2) enhancer [16]. This synergy could also be induced by activation of mitogen activated protein kinase (MAPkinase) pathways and the effects of capsaicin on ECR2-TAC1prom interaction could be blocked by antagonism of mitogen-activated protein kinase kinase (MEK kinase) [16]. We also revealed that stimulation of sensory neurones by capsaicin triggers ECR2- TAC1prom synergy in larger diameter sensory neurones; an expression of SP associated with hyperalgesia [16]. However, we were unable to determine whether the effects of capsaicin were cell autonomous or cell non- autonomous i.e. were the mechanisms controlling the up-regulation of TAC1prom only found within TRPV1 expressing cells or did these mechanisms involve communication between cells. Furthermore, if the effects of capsaicin are non-cell autonomous it is important to determine the communication pathways that induce ECR2-TAC1prom activity in large diameter neurones. In addition, because TRPV1 activation depolarises sensory neurones we also asked how specific the TRPV1-ECR2-TAC1prom relationship was and whether other methods of cell depolarisation such as potassium induced depolarisation, could induce ECR2-TAC1prom synergy [17,18].

Bacterial LPS has also been shown to upregulate the expression of TAC1 [5,6] and deletion of the TAC1 gene in mice reduces the effects of bacterial sepsis and LPS induced organ damage [19]. LPS is a potent activator of both the inflammatory response and the immune response through its receptor, Toll Like Receptor 4 (TLR4) [20]. It has been shown previously that activation of the TLR4 receptor triggers MAPkinase or NFκB dependent pathways [21]. However, it is not known whether TLR4 is expressed on sensory neurones, whether the MAPkinase of NFκB pathways modulate the effects of LPS on TAC1 expression, whether the effects of LPS is cell autonomous or whether the ECR2 enhancer is required for TAC1prom activation by LPS.

Using primary cell culture and transgenic explant culture we examined the mechanisms through which capsaicin, potassium mediated cellular depolarisation and LPS modulate TAC1prom activity within sensory neurones. Our findings demonstrate surprising diversity in the mechanisms modulating the activity of the TAC1 gene in sensory neurones. Identifying this diversity, as well as identifying of the components of the different mechanisms involved, will have an important baring on the future development of more targeted inflammatory pain therapies and will represent an important step in understanding the processes that regulate gene expression during the inflammatory response.

### Primary cell culture and transfection

Neonatal rat dorsal root ganglia (DRGs) were cultured as described previously [22] except for the following modifications. Following dissociation, DRG's neurons were re-suspended in Amaxa transfection solution. 100 μl of the cell suspension was added to a microcentrifuge tube containing 2 μg of plasmid DNA before being placed in an Amaxa cuvette and transfected using a nucleofector device set at program G-013. 100 ul of culture media (consisting of Hank's F12 meida (Gibco), putrescine, transferrin, BSA, progesterone, sodium selenite, FGF (20 μg/ml), insulin (100 μg/ml) and antibiotics) was added to the transfected cell suspension before being transferred into a culture dish containing 0.3 ml of culture media. Cells were left in the incubator and allowed to adhere to poly-L-lysine/laminin coated glass cover slips. Immediately after Amaxa transfection with DNA DRG neurons were cultured for 24 hours in a solution containing respective agonists or antagonists (See below). After culture, cells were fixed with 4% paraformaldahyde and expression of the LacZ gene (βgal) was visualised by staining with X-gal stain (5 mM K_3_Fe(CN)_6_; 5 mM K_4_Fe(CN)_6_; 2 mM MgCl_2_; 1 mg/ml X-gal) for two hours. The number of blue DRG neurons as a percentage of the total number of neurons was assessed by cell counting on an inverted DIC microscope. Proportions are adjusted relative to a control plasmid containing the CMV promoter that was transfected at the same time to normalise transfection efficiencies between different batches of rat neonates.

### Transgenic DRG explant analysis and immunocytochemistry

Whole DRG explants were dissected from ECR2-TAC1prom-LacZ transgenic mouse neonates and placed in the same culture conditions as described above. These explants were then treated with agonists or antagonists for 24 hours (see below), fixed in 4% paraformaldehyde and incubated with 30% sucrose in optimal cutting temperature (OCT) media overnight. Sections were cut with a cryostat to 10 μm thickness, permeabilised with 0.1% SDS for 5 mins then incubated in 10% foetal calf serum in tris-buffered saline with 1% triton (TBST) for 10 mins. Sections were washed 3 times for 5 mins in TBST and treated sequentially in primary antibodies overnight (rabbit-anti-βGal, 1:200; rat-anti-Substance P, 1:200 (AbCam) Goat-anti Toll-Like-Receptor 4 (TLR4) or rat anti-NK1 (1:200, Santa Cruz Biotechnology). Antibodies were visualised by incubation with the appropriate secondary antibody (diluted to 1:250) for 40 mins at room temp (goat-anti-rat Texas red, donkey-anti-Rabbit ALEXA 488 or Donkey-anti-goat ALEXA 488, all from Molecular Probes).

### Agonists and antagonists

Lipopolysaccharide, purchased from Sigma-Aldrich, was dissolved in PBS and used at a concentration of 10 μg/ml [23]). Capsaicin, purchased from Sigma-Aldrich, was dissolved in EtOH and used at a concentration of 10 μM [24]. Forskolin, obtained from Sigma, was dissolved in DMSO and used at a concentration of 10 μM [25]. [Sar9, Met(O2)11]-Substance P was obtained from Tocris, dissolved in H_2_O and used at a concentration of 100 nM as recommended by Tocris. KCl was obtained from Tocris, dissolved in H_2_O and used at a concentration of 30 mM [26].

#### Constructs

Details of the production of the constructs used in the current study have been described previously [16].

#### Image capture and analysis

Images were captured on Qicam monochrome camera mounted on a Nicon Eclipse 400 fluorescence microscope and processed using Improvision Velocity software v4.1. Figures were assembled on Adobe Photoshop CS3 software.

#### Statistical analysis

All experiments were repeated a minimum of three times on separate dates using separate groups of animals (n ≥ 3). Two tailed student t-tests were used to test the significance of data derived from primary cell cultures. Unpaired t-tests were used on data derived from transgenic explants studies to test the significance of the changes in the proportion of expressing cells within a specific cell diameter range. Statistical analysis was done using Microsoft Excel.

## Results

### Induction of TAC1 by capsaicin does not occur in all TRPV1 expressing cells

**Figure 1:**
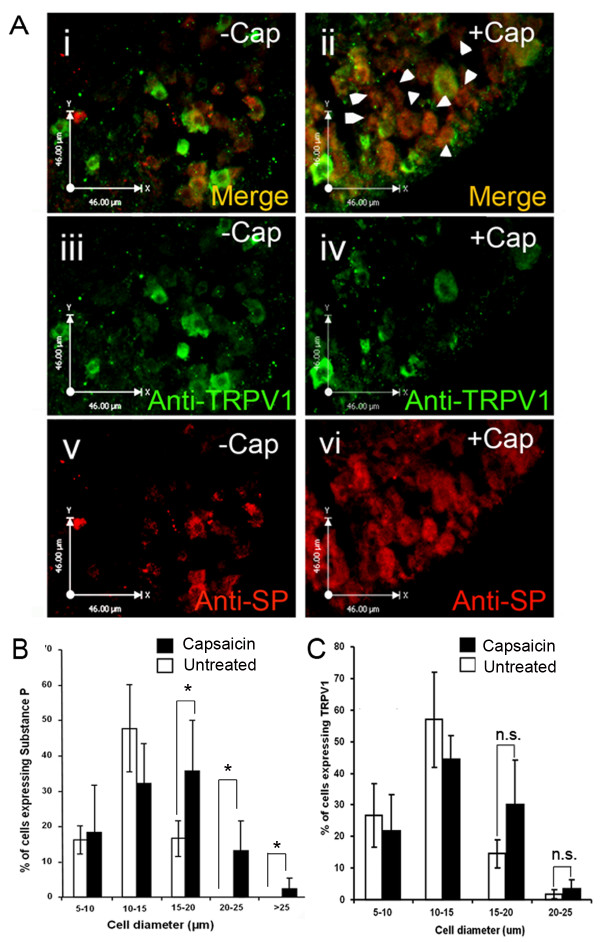
**Induction of TAC1 by capsaicin does not occur in all TRPV1 expressing cells**. fluorescent immunohistochemical analysis of 10 μm sections from mouse neonate DRG explants with (**iii **and **iv**) anti-TRPV1 and (**v **and **vi**) anti-SP antibody (**i, iii, v**) before and (**ii, iv, vi**) after 24 hours incubation with 10 μM capsaicin. **Ai **and **ii **represent merged images showing cellular co-localisation of TRPV1 and SP (yellow). White arrow heads highlight cells that express SP but not TRPV1 after capsaicin treatment. **B**, and **C**, graphical analysis of the size distribution (in microns) of neurones within DRG explants expressing (B) SP and (**C**) TRPV1 before (White bar) and after (black bar) capsaicin treatment (n = 3, total number SP/TRPV1 immunostaining cells counted and measured = 284, *; *p *< 0.05).

In our previous study we demonstrated perfect co- expression between the ECR2-TAC1prom-LacZ transgene and the SP peptide in larger diameter neurones following treatment of transgenic DRG explants with capsaicin [16]. In order to determine whether capsaicin induction of SP was cell autonomous (occured within the same cell) or non-cell autonomous (required cell-cell communication) we examined SP and TRPV1 co-expression using immunohistochemistry of mouse neonate DRG explants that had been exposed to 10 μM capsaicin for 24 hours. These DRG explants were then cryosectioned and fluorescent immunohistochemistry was carried out using antisera raised against TRPV1 and SP (Figure 1A). As described previously [16] immuno-positive cells were counted and their diameters were measured. The proportions of cells expressing either SP or TRPV1 are displayed in Figure 1B and 1C respectively. Consistent with our previous study [16] we were able to see a significant increase in the proportion of large diameter DRG neurones expressing SP following treatment with capsaicin (Figure 1A and 1B). However, we were unable to detect a correspondingly significant up-regulation in either the size or numbers of cells expressing the TRPV1 protein (Figure 1A and 1C). In addition, we observed a number of larger diameter neurones that expressed the SP peptide following capsaicin induction but did not express the TRPV-1 protein (Figure 1A, white arrows).

### The ECR2-TAC1prom transgene can be activated by cell depolarization

We have previously used luciferase assays to determine how TAC1prom responds to various signalling antagonists and agonists and its functional relationship with other regulatory elements [16]. However, although luciferase assays are very sensitive in showing changes in the expression of genes from large population of cells they are limited in their ability to give information on either the identity of these cells in mixed populations or on the numbers of cells expressing the reporter construct. This is an important consideration as previous studies have shown that, in addition to increasing levels of gene expression [7-10] capsaicin also increases the expression of the TAC1 gene in larger diameter neurones [3,4,27-31]. In order to address this problem we have previously combined luciferase assays with cell counting assays using the LacZ gene as the reporter. For example, using luciferase assays in heterogeneous primary DRG culture we show that upregulation of the activity of TAC1prom by capsaicin or the MAPkinase agonist angiotensin only occurs in the presence of the remote ECR2 enhancer [16]. These quantitative observations were supported by parallel experiments carried out with a LacZ marker which showed that in the presence of ECR2, TAC1prom responded to angiotensin and capsaicin by driving the expression of the LacZ marker in significantly greater numbers of cells [16].

**Figure 2:**
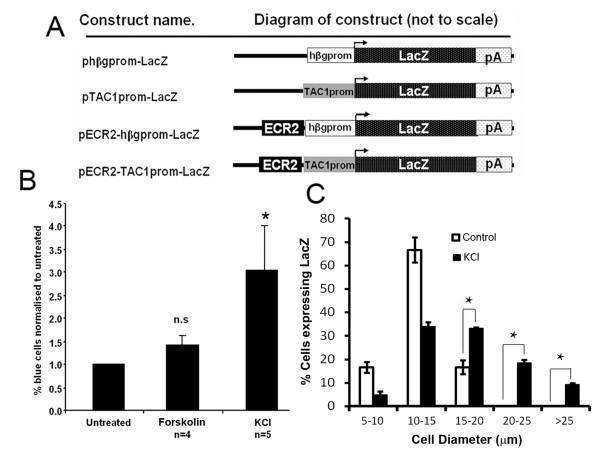
**The ECR2-TAC1prom transgene can be activated by cell depolarisation**. **A**, Diagramatic representation (not to scale) demonstrating the linear relationships of the components of each of the different constructs used in the current study. Construction of these reporter vectors has been previously described[16]. pA; SV40 polyadenylation sequence, lacZ; gene encoding β galactosidase marker protein, hβgprom; human beta globin promoter, TAC1prom; TAC1 promoter, ECR2; evolutionary conserved region 2, bent black arrow; indicates the transcriptional start site of the LacZ marker gene. **B; **bar graph demonstrating the proportion of primary DRG neurones that express βgal (as assayed using X-gal) following their transfection with pECR2-TAC1prom-LacZ and cultured in the presence of forskolin or KCl (n > 3, *;p < 0.05, n.s., not significant). Proportions are adjusted relative to a control plasmid containing the CMV promoter that was transfected at the same time to normalise transfection efficiencies. **C**, graphical analysis of the size distribution (diameter in microns) of neurones within ECR2-TAC1prom-LacZ transgenic DRG explants showing the proportion of cells expressing the β-gal before and after treatment with 30 mM KCl demonstrating a shift in the proportion of larger diameter cells expressing SP and the receptor (n = 3, No cells counted/measured = 201, *; p < 0.05).

Because activation of TRPV1 causes cellular depolarisation we wanted to test the specificity of the relationship between TRPV1 and ECR2-TAC1prom and to determine if potassium depolarisation of primary DRG neurones transformed with the ECR2-TAC1prom-LacZ construct (See Figure 2A) could increase the numbers of neurones expressing the reporter construct. We transfected primary DRG neurone cultures with the ECR2-TAC1prom-LacZ construct and incubated these cultures in the presence or absence of a 30 mM solution of KCl. Following staining with X-gal we then counted the number of cells that expressed the transgene and were able to detect a significant increase in the numbers of cells expressing βgal compared to untreated DRG neurones or neurones treated with forskolin which did not appear to have any significant effect (Figure 2B).

We also recovered intact DRG explants from neonate mice transgenic for the ECR2-TAC1prom-LacZ construct and cultured these cells in the presence and absence of 30 mM KCl [26]. We detected a significant increase in the number of cells with a diameter greater that 15 microns expressing the transgene mirroring the effects previously observed using capsaicin and angiotensin [16] (Figure 2C).

### Capsaicin can induce the expression of the NK1 receptor in DRG neurones that also express SP

**Figure 3:**
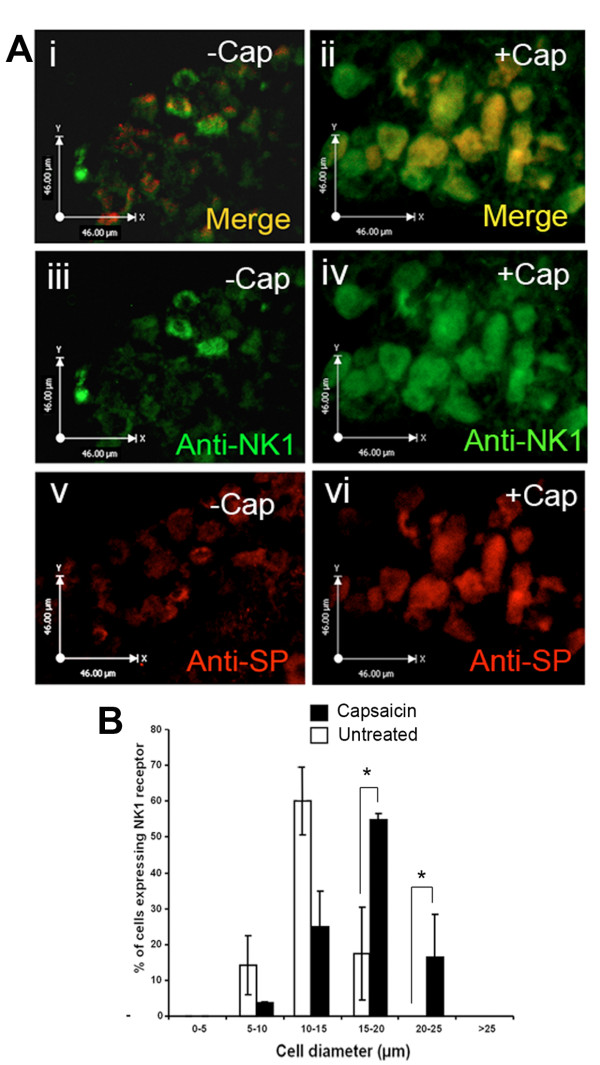
**Capsaicin can induce the expression of the NK1 receptor in DRG neurones that also express SP**. **A**. fluorescent immunohistochemical analysis of 10 μm sections from mouse neonate DRG explants with (**iii **and **iv**) anti-NK1 and (**v **and **vi**) anti-SP antibody (**i, iii, v**) before and (**ii, iv, vi**) after 24 hours incubation with 10 μM capsaicin. **Ai **and **ii **represent merged images showing cellular co-localisation of NK1 and SP (yellow). **B**, graphical analysis of the size distribution (in microns) of neurones within mouse neonate DRG explants showing the proportion of cells expressing both SP and NK1 expression before and after capsaicin treatment demonstrating a shift in the proportion of larger diameter cells expressing SP and the receptor (n = 3, No cells counted/measured = 242, *; p < 0.05).

It is a matter of contention that the NK1 receptor, which is accepted to be the main receptor of the SP neuropeptide, is expressed in DRG neurones [32,33]. However, a number of studies have since provided evidence of the expression of the receptor on the cell bodies of DRG neurones [34-38]. For example NK1 expression was detected in small DRG neurones by *in-situ *hybridisation and its activity was detected by electrophysiology in DRG cells using specific NK1 antagonists [37]. Furthermore, the expression of NK1 receptors is up-regulated in spontaneously hypertensive rats [35] and in DRG neurones exposed to prostaglandins [34]. In addition, activation of NK1 receptors in DRG culture potentiates the effects of TRPV1 and blockade of these NK1 receptors reduced the ability of TRPV1 activation to cause hyperalgesia and pain [38]. Other studies have further suggested the presence of an NK1-TAC1 autocrine loop in a number of different cells including DRG [38-42]. We explored the possibility that, in addition to the activity of TAC1prom, capsaicin treatment might also affect the expression of the NK1 receptor. We cultured mouse neonate DRG explants for 24 hours in the presence of 10 μM capsaicin and carried out fluorescent immunohistochemistry using antisera raised against the NK1 protein and SP (Figure 3A). As before, we observed a significant increase in the numbers of larger diameter neurones expressing SP following capsaicin treatment (Figure 3A). In addition, we found a similar increase in the number and diameter of cells expressing NK1 (Figure 3A and 3B). Most significantly, and in contrast to the expression of TRPV1, NK1 expression was found in all of the cells that expressed the transgene following induction by capsaicin (Figure 3A).

### Activation of the NK1 receptor induces expression of the ECR2-Tac1prom-LacZ transgene

**Figure 4:**
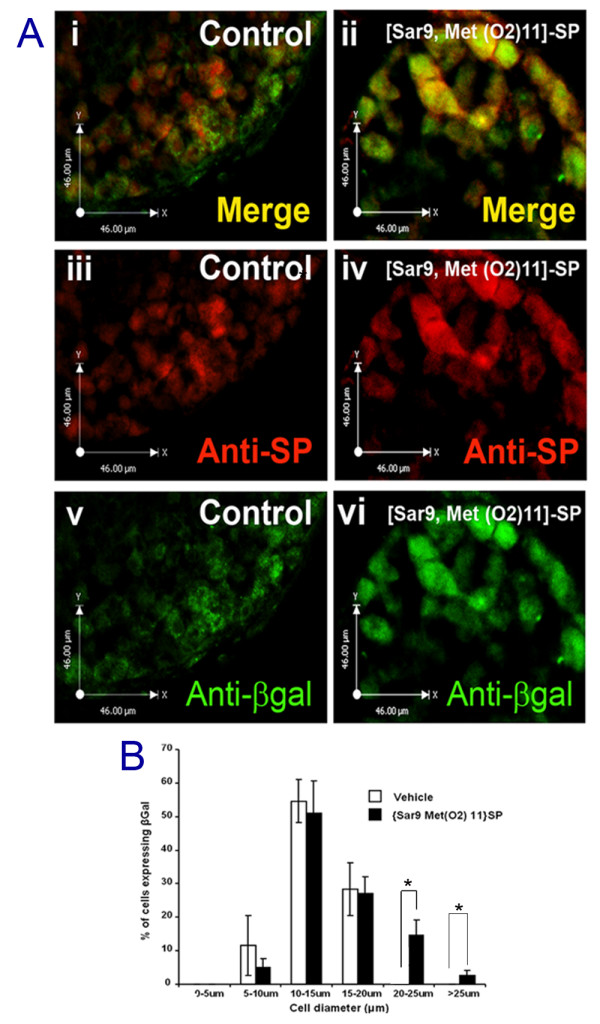
**Activation of the NK1 receptor induces expression of the ECR2-Tac1prom-LacZ transgene**. **A**. Fluorescent immunohistochemical analysis of 10 μm sections from ECR2-TAC1prom-LacZ transgenic DRG explants with (**iii **and **iv**) anti-SP and (**v **and **vi**) anti-βgal antibody (**i, iii, v**) before and (**ii, iv, vi**) after 24 hours incubation with 100 nM NK1 agonist [Sar9, Met(02)11]-SP. **Ai **and **ii **represent merged images showing cellular co-localisation of NK1 and SP (yellow). **B **graphical analysis of the size distribution (in microns) of neurones within ECR2-TAC1prom-LacZ transgenic DRG explants showing the proportion of cells expressing both SP and βgal expression before and after [Sar9, Met(01)11]-SP treatment demonstrating a shift in the proportion of larger diameter cells expressing SP and the receptor (n = 3, No cells counted/measured = 407, *; p < 0.05).

We provide evidence that expression of the NK1 receptor is induced by capsaicin in larger diameter DRG neurones that also express SP. We next addressed the possibility that activation of the NK1 receptor could activate the expression of the ECR2-TAC1prom transgene. To achieve this we treated ECR2-TAC1prom-LacZ transgenic DRG explants in the presence of the NK1 agonist [Sar9, Met(O2)11]-SP. Following 24 hours incubation with this agonist the expression of both SP and the βgal protein were examined using fluorescence immunohistochemistry (Figure 4A and 4B). Cell counting and measurement showed a significant up-regulation of the expression of both SP and the transgene in large diameter neurones (Figure 4A and 4B) in a manner reminiscent of treatment by capsaicin [16].

### Induction of TAC1prom by LPS is independent of ECR2

**Figure 5:**
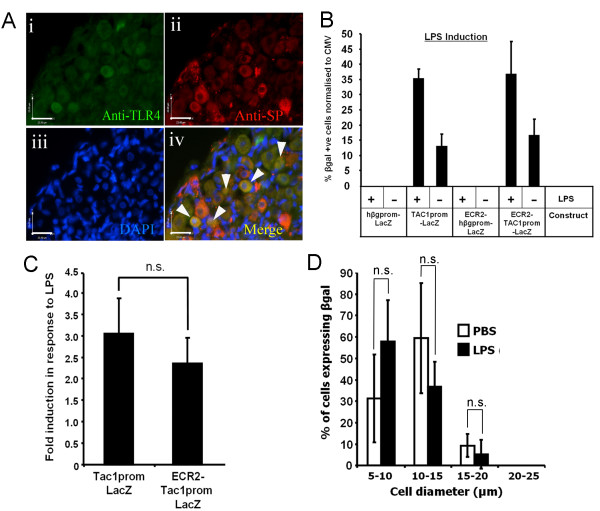
**Induction of TAC1prom by LPS is independent of ECR2**. **A**; immunohistochemical analysis of the expression of (**i**) TLR4 and (**ii**) SP and in mouse neonate DRG. **iv**; merged images and cells co-expressing SP and TRL4 are highlighted in yellow (white arrows). Scale bar = 23 microns. **B; **bar graph demonstrating the proportion of primary DRG neurones that express βgal (as assayed using X-gal) following their transfection with each of the constructs shown in figure 2A and cultured in the absence or presence of LPS (n > 3). Proportions are adjusted relative to a control plasmid containing the CMV promoter that was transfected at the same time to normalise transfection efficiencies. **C**; statistical analysis of data from the previous graph demonstrating that average induction rates for TAC1prom-LacZ by LPS is not significantly affected by the presence of ECR2. **D**; graphical analysis of the size distribution (in microns) of neurones within ECR2-TAC1prom-LacZ transgenic DRG explants analysing transgene expression following culture in the absence or presence of LPS (n = 3) demonstrating a lack of a shift in the proportion of larger diameter cells expressing βgal (n = 3, no. βgal cells counted and measured = 170).

A number of studies have shown that, in addition to capsaicin, SP expression can be induced by the bacterial cell wall extract LPS. These data support studies that show that deletions of the TAC1 gene in mice also reduce the effects of bacterial sepsis and LPS induced organ damage [6,19]. We explored the hypothesis that LPS used a similar regulatory pathway as capsaicin to up-regulate the activity of TAC1prom. Consistent with the role of LPS in inducing TAC1 gene expression we were able to co-localise the expression of the TLR4 receptor in a subset of cells of the DRG that also expressed SP (Figure 5A white arrows). In order to determine the effects of LPS on TAC1prom we transfected primary DRG neurones with the TAC1prom-LacZ, ECR2-hβg-LacZ and ECR2-TAC1prom-LacZ reporter plasmids (See Figure 2A) and treated these cultures with LPS. We demonstrate that LPS induced the activity of TAC1prom in a significantly greater number of cells than the untreated control (Figure 5B). However, these experiments also show that LPS induction of TAC1prom did not differ significantly in the presence or absence of ECR2 (Figure 5B and 5C). Furthermore, culture of ECR2-TAC1prom-LacZ transgenic DRG explants with LPS did not significantly change the size spectrum of the cells expressing the transgene (Figure 5D). Indeed no cells with a diameter greater than 15 microns expressed the transgene or SP following treatment with levels of LPS that were clearly shown to induce TAC1prom in primary neurones.

## Discussion

Neurogenic inflammation has been shown to play a critical role in the progression of diseases such as asthma, inflammatory bowel disease and arthritis [43]. Thus, gaining a better understanding of the mechanisms controlling the activity of TAC1prom has been a continuing priority since the discovery of the involvement of the TAC1 gene in neurogenic inflammation. We have previously demonstrated the novel requirement by TAC1prom for the presence of the ECR2 enhancer to respond appropriately to MEKKinase activation and noxious induction by capsaicin [16]. In the current study, we have used a combination of transgenic analysis, DRG explant culture and primary cell line transformation to further examine the effects of well characterised inflammatory mediators such as capsaicin and LPS on TAC1 promoter activity. We first demonstrated that the expression of TRPV1 did not change significantly in response to capsaicin and that many of the larger diameter neurones, that expressed substance-P in response to capsaicin, did not express TRPV1. These observations suggest that the induction of TAC1 promoter activity in larger diameter neurones by capsaicin is not cell autonomous. Furthermore, we demonstrate that depolarising DRG neurones with potassium was also able to induce expression of the transgene in large diameter neurones providing further evidence that the previously observed relationship between TRPV1 and the transgene depends on cell depolarisation. However, we also suggest that the induction of ECR2-TAC1prom synergism by potassium induced depolarisation might also activate voltage gated calcium channels (VGCC) that are also known to play a role in pain modulation in sensory neurones [44]. These observations support the hypothesis that TRPV1 is only one of many pathways that can stimulate synergy between ECR2 and the TAC1prom.

Because a number of previous studies have reported the expression of NK1 in these neurones, and also report on the possibility of an NK1-TAC1 autocrine loop, we next explored the possibility that the NK1 receptor may be involved in the induction of TAC1prom in DRG neurones [34,35,37,38]. In support of these observations we were able to show a parallel induction of NK1 and SP in larger diameter DRG neurones following capsaicin treatment. Significantly, we were also able to observe that treatment of ECR2- TAC1prom-LacZ transgenic DRG explants with an NK1 agonist triggered the expression of the transgene in large diameter neurones in a similar way to capsaicin. These observations are consistent with the proposition that stimulation of large diameter neurones with SP can induce the expression of SP within these neurones and induce an autocrine loop. The presence of an TAC1 gene driven autocrine loop in these large diameter neurones suggests a possible mechanisms for the induction and maintainance of hyperalgesia which is associated with the expression of SP in large diameter neurones[3,4]. However, it must be recognised that [Sar9, Met(O2)11]-SP can also bind a number of other receptors, including NK2 and NK3, although with greatly reduced affinity [45]. Therefore, whilst there is a strong case for the involvement of NK1 in the maintenance of TAC1prom activity in large diameter neurones following capsaicin treatment we are also aware that NK1 may not be the only receptor responsible and that redundancy with other receptors, such as NK3, is a possibility. In addition, although we present evidence for the presence of an NK1-ECR2-TAC1prom driven autocrine loop in the perpetuation of SP expression in large diameter neurones there is the possibility that another pathway, which does not involve NK1 signalling, is involved in the initial up-regulation of the NK1 gene expression in large diameter neurones

Following stimulation with KCL or capsaicin it was observed that the expression of the ECR2-TAC1prom-LacZ transgene or the NK1 receptor respectively were observed in significantly lower proportions of small diameter neurones. However, we have little evidence that the numbers of small diameter neurones expressing these markers decrease significantly after treatment. Therefore, these observations do not constitute a significant change in the numbers of cells expressing the respective marker but instead reflect a reduction in the overall proportion of small diameter cells expressing the marker as a result of the increased proportion of expressing larger diameter neurones.

**Figure 6:**
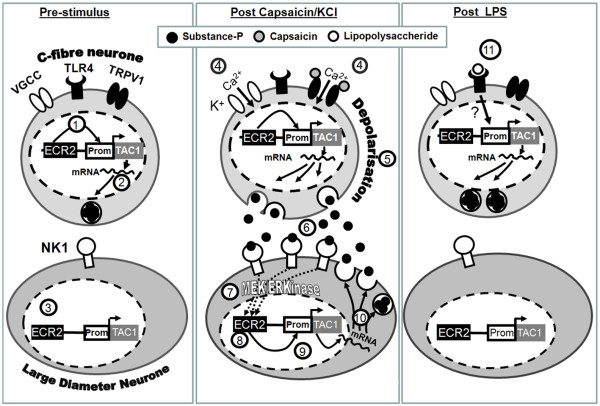
**Diagrammatical representation of the hypothetical genomic and cellular events that affect the activation of TAC1prom in sensory neurones treated with KCl, capsaicin or LPS**. This model is based on the conclusions of the current study and the literature. See the discussion section in the main text for a detailed description.

Based on these observations and those of other studies described above we present one possible model of the mechanisms regulating TAC1 promoter activity in sensory neurones in the absence or presence of capsaicin and potassium (see Figure 6). We propose that, prior to stimulation by capsaicin, LPS or potassium TAC1prom is only active in smaller diameter C-fibre neurones (Figure 6 step 1 and 2) and ECR2 is unable to promote activity of TAC1prom in larger diameter neurones prior to cell depolarisation (Step 3). However, following activation of TRPV1 ion channels with capsaicin or activation of VGCC's with potassium driven depolarisation (step 4) SP containing c-fibre neurones are depolarised and release SP (step 5 and 6). Following its release, SP then binds receptors that likely include the NK1 receptor on neighbouring cells (Step 6) triggering an autocrine loop (steps 7-10) that results in a parallel induction of TAC1prom and higher expression of NK1. These observations, when combined with those from our previous study [16] suggest the fascinating possibility that the presence of an autocrine loop in large diameter neurons may contribute to the perpetuation of hyperalgesic symptoms. Indeed, such an autocrine loop has been previously suggested in sensory neurons before [38]. It will be interesting to determine the mechanisms that allow induction of TAC1prom in larger diameter neurones by ECR2. Whilst it is possible that the function of ECR2, and the MAPkinase activated transcription factors interacting with it [16], is to directly enhance the activity of RNA polymerase II at TAC1prom it is also possible that an unidentified MEK/ERkinase activated transcription factor allows RNA polII activity at TAC1prom by removing the repressive effects of REST/NRSF known to bind at TAC1prom [46,47].

LPS was also shown to increase the numbers of primary neurones in which TAC1prom was active. However, in contrast to angiotensin and capsaicin [16] induction of TAC1prom by LPS did not depend on the presence of ECR2. In addition, we were unable to detect any evidence for the upregulation of the TAC1prom transgene in neurones greater that 15 microns in diameter in DRG explants exposed to LPS. Our data suggests that activation of TAC1prom by LPS is via an unidentified cell autonomous mechanisms that does not require the presence of ECR2 (step 11). We have previously shown that angiotensin II is unable to activate TAC1prom in the absence of ECR2 [16] Although LPS binding to TLR4 has been shown to activate MAPkinase pathways the lack of requirement by LPS for ECR2 in the induction of TAC1prom, in combination with the inability of MAPkinase to induce TAC1prom in the absence of ECR2, suggests that LPS does not control TAC1prom through MAPkinases. Thus, by deduction, LPS is more likely to modulate TAC1prom activity through NFκβ than MAPkinase pathways [21]. In keeping with this hypothesis there have been previous reports of highly conserved NFκβ binding in TAC1 exon 1 [48] and we have used bioinformatics to detect the presence of NFκβ binding sites close to the human TAC1 transcriptional start site (data not shown).

Despite evidence showing that LPS and capsaicin can activate TAC1 expression and inflammation the current study suggests a distinction between the signal transduction pathways that control either capsaicin or LPS activation of TAC1prom. In hindsight, the basis for these differences make sense as, despite both being able to activate the promoter, they are both known to operate through different signalling pathways [13,21]. Further analyses of the influence of NFκβ on activation of TAC1prom in sensory neurones exposed to LPS will be very exciting.

## Conclusions

The current study sheds light on the fascinating diversity of regulatory mechanisms that control the activity of TAC1prom during challenges by inflammatory mediators such as LPS and capsaicin and provides further evidence for the existence of an auto-regulatory loop that controls the expression of the TAC1 gene [34,35,37,38]. Gaining a better understanding of the diverse mechanisms that control the expression of TAC1prom during the inflammatory response will take us a step nearer to the development of more specific and novel therapies for the treatment of conditions such as hyperalgesia and inflammatory pain.

## Competing interests

The authors declare that they have no competing interests.

## Authors' contributions

LS and ML carried out all primary cell transfection and DRG explants studies. ML, AM and SD carried out all the cloning of the constructs used. RR and AM conceived of the study, and participated in its design and coordination. AM carried out the production of the transgenic lines. All authors have read and approved the final version of the manuscript.

## References

[B1] Zimmer A Zimmer AM Baffi J Usdin T Reynolds K Konig M Palkovits M Mezey E Hypoalgesia in mice with a targeted deletion of the tachykinin 1 gene Proc Natl Acad Sci USA 1998 95 2630 2635 10.1073/pnas.95.5.2630 9482938 PMC19441

[B2] Hokfelt T Pernow B Wahren J Substance P: a pioneer amongst neuropeptides J Intern Med 2001 249 27 40 10.1046/j.0954-6820.2000.00773.x 11168782

[B3] Neumann S Doubell TP Leslie T Woolf CJ Inflammatory pain hypersensitivity mediated by phenotypic switch in myelinated primary sensory neurons Nature 1996 384 360 364 10.1038/384360a0 8934522

[B4] Noguchi K Kawai Y Fukuoka T Senba E Miki K Substance P induced by peripheral nerve injury in primary afferent sensory neurons and its effect on dorsal column nucleus neurons J Neurosci 1995 15 7633 7643 7472514 10.1523/JNEUROSCI.15-11-07633.1995 PMC6578074

[B5] Huang HY Lai YL Lipopolysaccharide induces preprotachykinin gene expression Am J Respir Cell Mol Biol 2003 29 606 612 10.1165/rcmb.2002-0107OC 12738685

[B6] Ng SW Zhang H Hegde A Bhatia M Role of preprotachykinin-A gene products on multiple organ injury in LPS-induced endotoxemia J Leukoc Biol 2008 83 288 295 10.1189/jlb.0807575 17998302

[B7] Watanabe N Horie S Michael GJ Keir S Spina D Page CP Priestley JV Immunohistochemical co-localization of transient receptor potential vanilloid (TRPV)1 and sensory neuropeptides in the guinea-pig respiratory system Neuroscience 2006 141 1533 1543 10.1016/j.neuroscience.2006.04.073 16765524

[B8] Dinh QT Groneberg DA Peiser C Mingomataj E Joachim RA Witt C Arck PC Klapp BF Fischer A Substance P expression in TRPV1 and trkA- positive dorsal root ganglion neurons innervating the mouse lung Respir Physiol Neurobiol 2004 144 15 24 10.1016/j.resp.2004.08.001 15522699

[B9] Garrett NE Cruwys SC Kidd BL Tomlinson DR Effect of capsaicin on substance P and nerve growth factor in adjuvant arthritic rats Neurosci Lett 1997 230 5 8 10.1016/S0304-3940(97)00458-8 9259450

[B10] Donaldson LF McQueen DS Seckl JR Neuropeptide gene expression and capsaicin-sensitive primary afferents: maintenance and spread of adjuvant arthritis in the rat J Physiol 1995 486 Pt 2 473 482 7473211 10.1113/jphysiol.1995.sp020826 PMC1156535

[B11] Buchanan MM Hutchinson M Watkins LR Yin H Toll-like receptor 4 in CNS pathologies J Neurochem 2010 114 13 27 20402965 10.1111/j.1471-4159.2010.06736.x PMC2909662

[B12] Jara-Oseguera A Simon SA Rosenbaum T TRPV1: on the road to pain relief Curr Mol Pharmacol 2008 1 255 269 10.2174/1874467210801030255 20021438 PMC2802457

[B13] Ma W Quirion R Inflammatory mediators modulating the transient receptor potential vanilloid 1 receptor: therapeutic targets to treat inflammatory and neuropathic pain Expert Opin Ther Targets 2007 11 307 320 10.1517/14728222.11.3.307 17298290

[B14] Ueda H Molecular mechanisms of neuropathic pain-phenotypic switch and initiation mechanisms Pharmacol Ther 2006 109 57 77 10.1016/j.pharmthera.2005.06.003 16023729

[B15] Laird JM Roza C De Felipe C Hunt SP Cervero F Role of central and peripheral tachykinin NK1 receptors in capsaicin- induced pain and hyperalgesia in mice Pain 2001 90 97 103 10.1016/S0304-3959(00)00394-8 11166975

[B16] Shanley L Davidson S Lear M Thotakura AK McEwan IJ Ross RA Mackenzie A Long-Range Regulatory Synergy Is Required to Allow Control of the TAC1 Locus by MEK/ERK Signalling in Sensory Neurones Neurosignals 2010 in press 21160161 10.1159/000322010 PMC3718575

[B17] Morrison CF McAllister J Lyons V Chapman K Quinn JP The rat preprotachykinin-A promoter is regulated in PC12 cells by the synergistic action of multiple stimuli Neurosci Lett 1994 181 117 120 10.1016/0304-3940(94)90573-8 7898749

[B18] Mulderry PK Neuropeptide expression by newborn and adult rat sensory neurons in culture: effects of nerve growth factor and other neurotrophic factors Neuroscience 1994 59 673 688 10.1016/0306-4522(94)90186-4 7516508

[B19] Killingsworth CR Paulauskis JD Shore SA Substance P content and preprotachykinin gene-I mRNA expression in a rat model of chronic bronchitis Am J Respir Cell Mol Biol 1996 14 334 340 8600937 10.1165/ajrcmb.14.4.8600937

[B20] Bosshart H Heinzelmann M Targeting bacterial endotoxin: two sides of a coin Ann N Y Acad Sci 2007 1096 1 17 10.1196/annals.1397.064 17405910

[B21] Lu YC Yeh WC Ohashi PS LPS/TLR4 signal transduction pathway Cytokine 2008 42 145 151 10.1016/j.cyto.2008.01.006 18304834

[B22] Shewan D Berry M Cohen J Extensive regeneration in vitro by early embryonic neurons on immature and adult CNS tissue J Neurosci 1995 15 2057 2062 7891152 10.1523/JNEUROSCI.15-03-02057.1995 PMC6578146

[B23] Hou L Wang X PKC and PKA, but not PKG mediate LPS-induced CGRP release and [Ca(2+)](i) elevation in DRG neurons of neonatal rats J Neurosci Res 2001 66 592 600 10.1002/jnr.1249 11746379

[B24] Yang X Gong H Liu Z Liu H Wang H Li Z Similar and different effects of capsaicin and resiniferatoxin on substance P release and transient receptor potential vanilloid type 1 expression of cultured rat dorsal root ganglion neurons Methods Find Exp Clin Pharmacol 2010 32 3 11 10.1358/mf.2010.32.1.1444424 20383340

[B25] Dolphin AC Ca2+ channel currents in rat sensory neurones: interaction between guanine nucleotides, cyclic AMP and Ca2+ channel ligands J Physiol 1991 432 23 43 1653319 10.1113/jphysiol.1991.sp018374 PMC1181315

[B26] Agis-Torres A Ball SG Vaughan PF Chronic treatment with nicotine or potassium attenuates depolarisation-evoked noradrenaline release from the human neuroblastoma SH-SY5Y Neurosci Lett 2002 331 167 170 10.1016/S0304-3940(02)00881-9 12383923

[B27] Bulling DG Kelly D Bond S McQueen DS Seckl JR Adjuvant-induced joint inflammation causes very rapid transcription of beta- preprotachykinin and alpha-CGRP genes in innervating sensory ganglia J Neurochem 2001 77 372 382 10.1046/j.1471-4159.2001.00175.x 11299299

[B28] Noguchi K Morita Y Kiyama H Ono K Tohyama M A noxious stimulus induces the preprotachykinin-A gene expression in the rat dorsal root ganglion: a quantitative study using in situ hybridization histochemistry Brain Res 1988 464 31 35 3179743 10.1016/0169-328x(88)90015-0

[B29] Marchand JE Wurm WH Kato T Kream RM Altered tachykinin expression by dorsal root ganglion neurons in a rat model of neuropathic pain Pain 1994 58 219 231 10.1016/0304-3959(94)90202-X 7816489

[B30] Malcangio M Ramer MS Jones MG McMahon SB Abnormal substance P release from the spinal cord following injury to primary sensory neurons Eur J Neurosci 2000 12 397 399 10.1046/j.1460-9568.2000.00946.x 10651897

[B31] Pitcher GM Henry JL Nociceptive response to innocuous mechanical stimulation is mediated via myelinated afferents and NK-1 receptor activation in a rat model of neuropathic pain Exp Neurol 2004 186 173 197 10.1016/j.expneurol.2003.10.019 15026255

[B32] McCarson KE Krause JE NK-1 and NK-3 type tachykinin receptor mRNA expression in the rat spinal cord dorsal horn is increased during adjuvant or formalin- induced nociception J Neurosci 1994 14 712 720 8301359 10.1523/JNEUROSCI.14-02-00712.1994 PMC6576805

[B33] Szallasi A Farkas-Szallasi T Tucker JB Lundberg JM Hokfelt T Krause JE Effects of systemic resiniferatoxin treatment on substance P mRNA in rat dorsal root ganglia and substance P receptor mRNA in the spinal dorsal horn Brain Res 1999 815 177 184 10.1016/S0006-8993(98)01168-8 9878727

[B34] Segond von Banchet G Scholze A Schaible HG Prostaglandin E2 increases the expression of the neurokinin1 receptor in adult sensory neurones in culture: a novel role of prostaglandins Br J Pharmacol 2003 139 672 680 10.1038/sj.bjp.0705278 12788827 PMC1573877

[B35] Aline Boer P Ueno M Sant'ana JS Saad MJ Gontijo JA Expression and localization of NK(1)R, substance P and CGRP are altered in dorsal root ganglia neurons of spontaneously hypertensive rats (SHR) Brain Res Mol Brain Res 2005 138 35 44 10.1016/j.molbrainres.2005.03.015 15869822

[B36] Ackermann KH Adams N Adler C Ahammed Z Ahmad S Allgower C Amsbaugh J Anderson M Anderssen E Arnesen H Elliptic flow in Au+Au collisions at square root(S)NN = 130 GeV Phys Rev Lett 2001 86 402 407 10.1103/PhysRevLett.86.402 11177841

[B37] Li H Bao Y Zhao Z Expression of tachykinin receptors inXenopus oocytes injected with poly (A)(+) RNA from cat dorsal root ganglion Sci China C Life Sci 1998 41 139 145 10.1007/BF02882718 18726197

[B38] Zhang H Cang CL Kawasaki Y Liang LL Zhang YQ Ji RR Zhao ZQ Neurokinin-1 receptor enhances TRPV1 activity in primary sensory neurons via PKCepsilon: a novel pathway for heat hyperalgesia J Neurosci 2007 27 12067 12077 10.1523/JNEUROSCI.0496-07.2007 17978048 PMC6673346

[B39] Germonpre PR Bullock GR Lambrecht BN Van De Velde V Luyten WH Joos GF Pauwels RA Presence of substance P and neurokinin 1 receptors in human sputum macrophages and U-937 cells Eur Respir J 1999 14 776 782 10.1034/j.1399-3003.1999.14d08.x 10573219

[B40] Fontan JJ Cortright DN Krause JE Velloff CR Karpitskyi VV Carver TW Shapiro SD Mora BN Substance P and neurokinin-1 receptor expression by intrinsic airway neurons in the rat Am J Physiol Lung Cell Mol Physiol 2000 278 L344 355 10666119 10.1152/ajplung.2000.278.2.L344

[B41] Maghni K Michoud MC Alles M Rubin A Govindaraju V Meloche C Martin JG Airway smooth muscle cells express functional neurokinin-1 receptors and the nerve-derived preprotachykinin-a gene: regulation by passive sensitization Am J Respir Cell Mol Biol 2003 28 103 110 10.1165/rcmb.4635 12495938

[B42] Andersson G Danielson P Alfredson H Forsgren S Presence of substance P and the neurokinin-1 receptor in tenocytes of the human Achilles tendon Regul Pept 2008 150 81 87 10.1016/j.regpep.2008.02.005 18394729

[B43] O'Connor TM O'Connell J O'Brien DI Goode T Bredin CP Shanahan F The role of substance P in inflammatory disease J Cell Physiol 2004 201 167 180 15334652 10.1002/jcp.20061

[B44] Zamponi GW Lewis RJ Todorovic SM Arneric SP Snutch TP Role of voltage-gated calcium channels in ascending pain pathways Brain Res Rev 2009 60 84 89 10.1016/j.brainresrev.2008.12.021 19162069 PMC2692704

[B45] Gether U Johansen TE Schwartz TW Chimeric NK1 (substance P)/NK3 (neurokinin B) receptors. Identification of domains determining the binding specificity of tachykinin agonists J Biol Chem 1993 268 7893 7898 7681831

[B46] Quinn JP Bubb VJ Marshall-Jones ZV Coulson JM Neuron restrictive silencer factor as a modulator of neuropeptide gene expression Regul Pept 2002 108 135 141 10.1016/S0167-0115(02)00103-9 12220737

[B47] Spencer EM Chandler KE Haddley K Howard MR Hughes D Belyaev ND Coulson JM Stewart JP Buckley NJ Kipar A Regulation and role of REST and REST4 variants in modulation of gene expression in in vivo and in vitro in epilepsy models Neurobiol Dis 2006 24 41 52 10.1016/j.nbd.2006.04.020 16828291

[B48] Corcoran KE Rameshwar P Nuclear factor-kappaB accounts for the repressor effects of high stromal cell-derived factor-1alpha levels on Tac1 expression in nontumorigenic breast cells Mol Cancer Res 2007 5 373 381 10.1158/1541-7786.MCR-06-0396 17409218

